# Verification of Abbott 25-OH-vitamin D assay on the architect system

**DOI:** 10.1016/j.plabm.2017.01.001

**Published:** 2017-01-17

**Authors:** Katrina Hutchinson, Martin Healy, Vivion Crowley, Michael Louw, Yury Rochev

**Affiliations:** a School of Chemistry and NCBES, National University of Ireland, Galway, Ireland; b Sechenov First Moscow State Medical University, Institute for Regenerative Medicine, Russia; c Biomnis Ireland, Sandyford, Dublin 18, Ireland; d Biochemistry Department, St. James's Hospital Dublin, Ireland

## Abstract

**Objectives:**

Analytical and clinical verification of both old and new generations of the Abbott total 25-hydroxyvitamin D (25OHD) assays, and an examination of reference Intervals.

**Methods:**

Determination of between-run precision, and Deming comparison between patient sample results for 25OHD on the Abbott Architect, DiaSorin Liaison and AB SCIEX API 4000 (LC-MS/MS). Establishment of uncertainty of measurement for 25OHD Architect methods using old and new generations of the reagents, and estimation of reference interval in healthy Irish population.

**Results:**

For between-run precision the manufacturer claims 2.8% coefficients of variation (CVs) of 2.8% and 4.6% for their high and low controls, respectively. Our instrument showed CVs between 4% and 6.2% for all levels of the controls on both generations of the Abbott reagents. The between-run uncertainties were 0.28 and 0.36, with expanded uncertainties 0.87 and 0.98 for the old and the new generations of reagent, respectively. The difference between all methods used for patients’ samples was within total allowable error, and the instruments produced clinically equivalent results. The results covered the medical decision points of 30, 40, 50 and 125 nmol/L. The reference interval for total 25OHD in our healthy Irish subjects was lower than recommended levels (24–111 nmol/L).

**Conclusion:**

In a clinical laboratory Abbott 25OHD immunoassays are a useful, rapid and accurate method for measuring total 25OHD. The new generation of the assay was confirmed to be reliable, accurate, and a good indicator for 25OHD measurement. More study is needed to establish reference intervals that correctly represent the healthy population in Ireland.

## Highlights

• A verification of the new version of the Abbott Architect 25OHD assay has been performed.
• The prevalence of obesity in Ireland may be a contributing factor to low 25OHD levels.
• Vitamin D (25OHD) testing can be important for general health screening.

## 1 Introduction

Vitamin D deficiency (VDD) is a widespread condition that is said to affect about 1 billion people worldwide [1]. Recent studies have shown that VDD is not only associated with bone and severe liver and kidney disease; it also has important implications in many chronic illnesses, including cancer, diabetes mellitus, hypertension and asthma [2]. Vitamin D is a fat-soluble steroid prohormone that is mainly produced photochemically in the skin from 7-dehydrocholesterol. Two forms of vitamin D are biologically important – vitamin D3 (Cholecalciferol) and vitamin D2 (Ergocalciferol).

Both vitamins D3 and D2 can be absorbed from food and can be found in vitamin supplements, but it is estimated that only 10–20% of vitamin D is supplied through food [3]. Vitamin D is metabolised to the active hormone 1,25(OH)_2_-vitamin D (Calcitriol) through two hydroxylation reactions. The first of these occurs in the liver, converting vitamin D into 25-hydroxyvitamin D (25OHD). The second hydroxylation converts 25OHD into the biologically active 1,25(OH)_2_-vitamin D and occurs in the kidneys and in many other cells. Most cells express the vitamin D receptor (VDR) and about 3% of the human genome is regulated by the vitamin D endocrine system [3]. The vitamin D level is generally assessed by measuring the serum or plasma level of 25OHD. This has a circulating half-life of 2–3 weeks and is not influenced by changes in calcium and parathyroid hormone levels [4]. This major storage and circulating form is a reliable indicator of vitamin D status [5]. Low 25OHD concentrations are associated with secondary hyperparathyroidism, skeletal diseases such as rickets, and many chronic illnesses [2], [6].

A panel from the Institute of Medicine (IOM) of the National Academy of Sciences decided that, on the basis of skeletal anomalies, VDD can be defined as a serum 25OHD below 50 nmol/L [7]. A newly proposed definition of vitamin D sufficiency (>50 nmol/L) has proved to be controversial., [8], [9] mainly because it has a major impact on the clinical evaluation of vitamin D insufficiency. There is no current consensus on the optimal vitamin D levels for non-musculoskeletal health; therefore it is important to establish reference intervals among the general population – specifically, in different seasons.

Vitamin D tests are now widely included as a part of routine laboratory work. A significant increase in laboratory testing for 25OHD has resulted in the development and the implementation of new automated diagnostic approaches to keep pace with the volume of demand. In our laboratory we have looked for a rapid, reliable, fully automated and cost saving assay that would improve turnaround time for our clients.

Numerous methods have been developed to measure serum and plasma 25OHD concentrations. The first routine methods for assessing 25OHD were based on competitive protein binding. These have been supplanted by radioimmunoassay (RIA) and by chemiluminescent immunoassay (CLIA), which form the fundamental principles of several commercially available methods. Two main analytical techniques usually employed in the laboratories are competitive immunoassays and chemical methods. Among the immunoassays are the following: RIA, CLIA, enzyme immunoassay, electrochemiluminescence immunoassay, chemiluminescent microparticle immunoassay (CMIA) and competitive protein binding assay. The chemical methods are based on chromatographic separation, followed by non-immunological direct detection. They include direct high performance liquid chromatographic with ultraviolet (HPLC-UV) detection, and liquid chromatography combined with mass spectrometry (LC-MS). The principal difference between these methods is the ability of HPLC and LC-MS to quantify 25(OH)D2 and 25(OH)D3 separately [10]. Recently a C3-epimeric form of 25(OH)D3 has been detected which is unresolved in some LC-MS/MS assays but may add to the total result of 25OHD. The epimer, however, has been found primarily in neonatal samples and it has been suggested that it does not contribute significantly to overall measured 25OHD concentration [11], [12].

The Joint Committee for Traceability in Laboratory Medicine (JCTLM) recognizes isotope dilution liquid chromatography tandem mass spectrometry (ID-LC-MS/MS) as the reference method for vitamin D. Mass spectrometry is highly sensitive and specific and can analyse several related analytes in a single run, with potential for cost savings. The introduction of commercial validated assay kits, traceable standards and participation in external quality assessment schemes has substantially improved the quality of LC-MS/MS assays for 25(OH)D3. However, initial capital purchase of these instruments can be costly, they require highly trained staff, and they are currently better suited to larger laboratories.

In recent years, in vitro diagnostic companies have introduced new automated immunoassays for the measurement of 25(OH)D3, thus improving the laboratory's ability to cope with increasing demand.

Many publications have outlined the limitations of these immunoassays. Some have poor antibody specificity with cross-reactivity to other metabolites of Vitamin D, as well as a problematic extraction of the 25OHD form from the vitamin D-binding protein (DBP). Some assays also interact with matrix substances such as lipids, and there are notable variations in 25OHD determination between different assays [13], [14], [15], [16]. Until recently, standardisation and harmonisation between the various marketed vitamin D assays was poor. The introduction of a new traceable reference standard (National Institute of Standards and Technology Standard Reference material 972; NIST SRM 972), however, has improved both validation and calibration of 25OHD assays across different platforms.

In our laboratory, a Vitamin D assay was run and accredited to ISO 15189 on the DiaSorin Liaison instrument. When the CE-marked Abbott Architect 25OHD assay became available it had already been extensively validated by the manufacturers, so we did not feel that a full validation was indicated. In 2016 a new generation of the Abbott reagents came to the market, standardised to NIST SRM 2972.

The purpose of this study was: to confirm that the assay was performing in our laboratory to the manufacturer's standards; to establish any clinical difference between results obtained using the Liaison, Architect and LC-MS/MS methods; to calculate the uncertainty of measurement for the assay; to run External Quality Assessment samples to assess bias; and to compare reference interval study results with current 25OHD recommendations.

## 2 Materials and methods

### 2.1 Abbott Architect and DiaSorin Liaison Immunoassays

The original Architect (Abbott Diagnostics, Lake Forest, IL, USA) 25OH Vitamin D assay (product 3L52) is a delayed 1-step chemiluminescence microparticle immunoassay (CMIA) involving automated online pre-treatment with flexible assay protocols, which are known as Chemiflex. It uses microparticles coated with a polyclonal sheep anti-vitamin D IgG antibody, and a biotinylated vitamin D anti-biotin IgG acridinium-labelled conjugate complex for the quantitative determination of 25(OH)D2 and D3 in human serum and plasma. The measuring range for the original assay is 32.5–240 nmol/L. The new generation of the Architect 25OHD assay (product 5P02) is also a delayed 1-step competitive immunoassay, but is notably improved. The conjugate is added after microparticles have incubated with the sample, but there is no wash before the addition of conjugate, and conjugate fills the vacant sites on the microparticle antibody. The pre-treatment (8-anilo-1-naphalensulfonic acid; ANSA in assay specific diluent) is added at the same time as the microparticle (rabbit monoclonal anti-Human vitamin D IgG) reagent. The avidity and stability of the microparticle antibody for vitamin D is much higher than in the old generation reagent. The acridinium-labelled conjugate is also an improved, much simpler and more stable conjugate than the previous assay. The crossreactivity between the assays is similar: 105% with 25(OH)D3, 54% with 25(OH)D2 and very low with 3-epi-25(OH)D3 (1.3%). The new generation measuring range is 8.5–389.8 nmol/L. Both assays are Vitamin D Standardisation Programme (VDSP) certified and they successfully passed the performance criterion of±5% mean bias of the Centres of Disease Control (CDC) and University of Ghent Vitamin D2 and D3 Reference Method with an overall imprecision of <10% over the concentration range of 22–275 nmol/L for total 25-hydroxyvitamin D.

The DiaSorin (Dietzenbach, Germany) Liaison used in our laboratory for total 25OHD is a direct competitive chemiluminescent immunoassay with inter- and intra-assay coefficients of variation (CVs) of 6.8% and 7%, respectively. This method is reported to have relative crossreactivities with 25(OH)D3, 25(OH)D2, and 3-epi-25(OH)D3 of 100%, 104%, and less than 1%, respectively. The analytical measuring range is 10–375 nmol/L.

### 2.2 Mass spectrometry

The mass spectrometer vitamin D assays were performed in the Biochemistry Department of St. James's Hospital, Dublin, a fully accredited laboratory to ISO 15189.

The vitamin D assay was developed by Chromsystems GmbH (Munich, Germany; Catalogue No. 62000) for analysis by LC/MS. The method is CE marked and uses standards traceable to NIST SRM 972. The assay quality is monitored by participation in the Vitamin D External Quality Assessment Scheme (DEQAS). The reportable results for this test included the concentrations of 25(OH)D2, 25(OH)D3, and total 25OHD (calculated as the sum of 25(OH)D2 and 25(OH)D3). The analytical measuring range is 1.5–624 nmol/L.

The within-run coefficient of variation was 4.1% at 60.7 nmol/L for 25(OH)D2 and 3.4% at 27.7 nmol/L for 25(OH)D3. The between run imprecision was 5.8% at 42.6 nmol/L and 5.7% at 95.8 nmol/L for 25(OH)D2, 6.1% at 41.6 nmol/L, and 5.0% at 98.2 nmol/L for 25(OH)D3.

### 2.3 Other methods

For the reference intervals study all serum measures of total calcium, albumin, alkaline phosphatase (ALP), phosphate, total protein, bilirubin, alanine transaminase, aspartate aminotransferase, gamma-glutamyl transferase, urea and creatinine were performed, using commercially available diagnostic kits on the Abbott Architect ci8200 (Abbott Laboratories). The mean between-run and within-run CVs for these assays ranged between 1% and 5%.

### 2.4 Statistical softwares

We used EP Evaluator Release 9 software (Data Innovations, LLC, Southe Burlington, VT, USA), GraphPad Prism 5, version 5.01 (GraphPad Software, La Jolla, CA, USA) and Microsoft Excel 2010 for the statistical evaluation of results.

### 2.5 Samples

We used only serum samples for 25OHD determination. All the samples were treated according to our standard preanalytical procedure: after sampling, they were centrifuged at +4 °C at 3500 G, aliquoted within 1 h, and kept frozen at −20 °C until determination, as it has been shown that 25OHD is particularly stable [17]. For the examination of reference intervals samples were obtained from healthy Irish participants as part of their routine health and lifestyle screen. Method comparison studies were carried out on samples from patients (age range: 5–80 years) suffering from different diseases, specifically osteoporosis and asthma. As mentioned above, epimer form is not discussed because it is not relevant to our study [11], [12]. The values for total 25OHD varied from 2.5 to 223 nmol/L. We used serum specimens submitted to a laboratory for the measurement of total 25OHD from three Dublin hospitals (Connolly, The Adelaide and Meath, and St. James's). As vitamin D deficiency is very common in Ireland, we fortified some volunteers from the patients with softgel capsules of 2.000 IU cholecalciferol for 15 weeks daily (Best Formulations ®, City of Industry, CA, USA). All supplemented subjects signed an institutional review board-approved written informed consent form. The Hospitals Research Ethics Committees approved the study. Abbott 25OHD Quality Controls and Technopath Immunoassays Controls (Technopath Clinical Diagnostics, Ballina, Ireland) were used for the imprecision and uncertainty of measurement study.

### 2.6 Validation protocol

We evaluated the imprecision based on guidance from the National Committee for Clinical Laboratory Standards (NCCLS) Protocol EP5-A2 [18]. For between-run imprecision, 3 levels of Technopath Immunoassays Controls were analysed for twenty days, using one instrument and the same lot of reagents. A method comparison was assessed between patient sample results run on the DiaSorin Liaison, AB SCIEX API 4000 liquid chromatography tandem mass spectrometer and the Architect, using EP Evaluator software (release 9, build 4–457). The two-instrument comparison method (based on allowable total error) was used to determine clinical equivalence of the results from the instruments. Clinical equivalence was defined as follows: two methods were deemed clinically equivalent if the difference between them was less than the allowable total error for each specimen. It does not matter if there is a bias, provided that the bias is within allowable error. All methods were run according to the manufacturer's instructions.

We established uncertainty of measurement for both the old and new generations of the Abbott method, based on our internal standard operating procedure, using calculations from the National Physical Laboratory [19]. Abbott had provided us with their uncertainty data, but these were irrelevant to our study and we did not use them in our calculations [20]. The within-run variation (uwr) was calculated from the mean and standard deviation (SD) of repeated measurements of a single sample in single run. The standard uncertainty (u) was calculated using the formula: $u=\frac{s}{\sqrt{n}}$, where s= the SD, and n = the number of measurements. The between-run variation (ubr) was calculated from the mean, and the SD obtained from the daily control runs. To calculate the total uncertainty of measurement (u_c_) for 25OHD we used summation in quadrature (root sum of the squares) of the standard uncertainties: Combined uncertainty $u_{c}=\sqrt{{\left(\mathrm{uwr}\right)}^{2}+{\left(\mathrm{ubr}\right)}^{2}}$.

To calculate the 95% confidence interval (CI), this combined uncertainty was multiplied by a “coverage factor” (k) of 2. The result is known as the “expanded uncertainty”.

To calculate Uncertainty of Measurement (UOM), one sample, with a 25OHD concentration of 50 nmol/L, was run 20 times to calculate within-run imprecision. For between-run imprecision, controls with concentrations of 67 and 57 nmol/L for the old and new generation assays, respectively, were run daily for more than 30 days. We calculated an expanded uncertainty with k=2. (Table 1) The precision protocols differed for the old and new assays, due to differences in the lot of the Quality Controls used and the number of samples analysed.

**Table 1:** Uncertainty of measurement (UOM) for old and new generation total 25OHD Architect methods (see text).

**Test**	**N**	**Value (nmol/L)**	**uwr (nmol/L)**	**ubr (nmol/L)**	**UOM (k=2) Absolute value (nmol/L)**	**UOM (%)**
**25OHD (3L52, OLD)**	172	67	0.335	0.28	0.87	1.30
**25OHD (5P02, NEW)**	66	57	0.266	0.36	0.98	1.72

N= number of samples, uwr=within-run uncertainty, ubr=between-run uncertainty,

k= coverage factor.

Serum samples from 210 healthy individuals were assayed to assess reference intervals for 25OHD and to verify the reference interval used with the Liaison method. All the patients were carefully screened for any chronic illnesses. The samples were analysed for total calcium, albumin, ALP, phosphate, urea, creatinine and liver enzymes. We studied only patients whose results were entirely within the reference ranges for the assays. Body mass Index (BMI) was calculated using participants’ weight and height (weight/height^2^). We also acquired information from healthy adults relating to their age, sex, race, medications taken, and vitamin D supplements. Reference interval data were analysed according to the National Committee for Clinical Laboratory Standards guideline for determining reference values [21]. Data were analysed using EP Evaluator Nonparametric Method (CLSI C28-A) [21] for calculating a Reference Interval. This method makes no assumption about the shape of population distribution. The central 95% of the data were taken as the reference interval for 25OHD.

## 3 Results

For between-run imprecision, Stockl et al. [22] suggest a maximum CV of 10% for 25OHD analysis. The manufacturers claim 4.6% for their low control, 3.0% for medium, and 2.8% for the high level. Our instrument showed a CV of 6.2% for our 40 nmol/L low control, 3.9% for the 67 nmol/L medium control and 4% for 100 nmol/L high level for Architect 25OHD Old assay (3L52). For the new generation of the reagents (5P02) we found a CV of 5.6% for our 36 nmol/L low control, 5% for 57 nmol/L medium and 5% for 113 nmol/l high level.

**Fig. 1:**
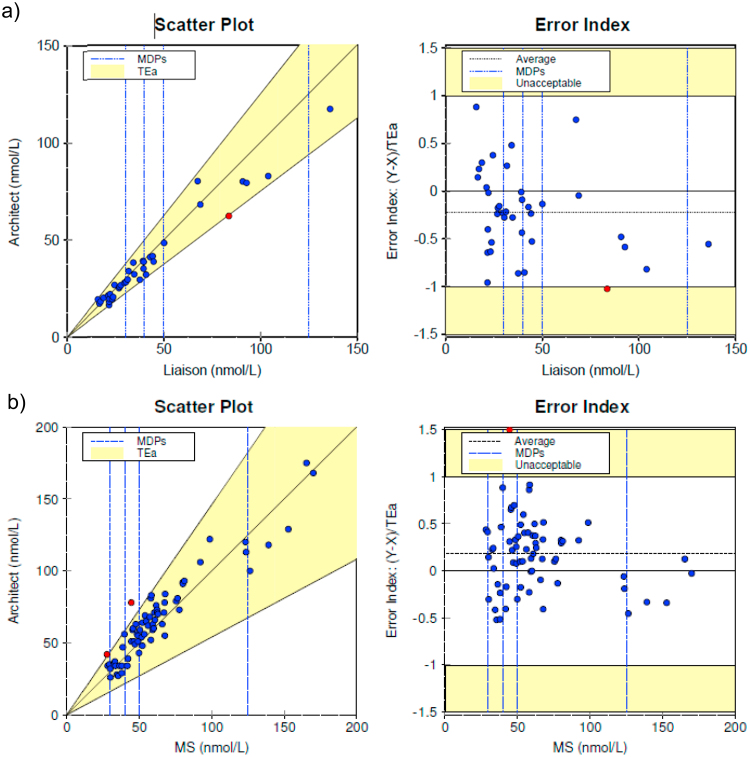
Two instrument comparisons between (a) Liaison and old Architect 25OHD (3L52) and (b) old Architect 25OHD method and LC-MS/MS (MS.) The regression line for 37 samples (a), calculated according to Deming, is indicated (25OHD old Architect nmol/L =0.85 x (25OHD Liaison) nmol/L+3.11nmol/L, r^2^=0.98). The regression line for 69 samples (b), calculated according to Deming, is indicated (25OHD old Architect nmol/L =0.96 x (25OHD MS) nmol/L+6.73 nmol/L, r^2^=0.94). The difference between the methods was within total allowable error (TEa) of 46%. The instruments produced clinically equivalent results. Results covered the medical decision points (MDPs) of 30, 40, 50 and 125 nmol/L.

The instrument comparison data were analysed for clinical equivalence by EP Evaluator using the Two Instrument Comparison module. An Allowable Total Error (TEa) was used in accordance with the recommendations of Stockl et al. [22] .These calculations were “derived from the evaluation criteria set by the Advisory Panel of DEQAS” [23] using a common proficiency testing programme, i.e. 46%. The difference between the Liaison and Architect instruments for 36 of 37 (97.3%) samples was within TEa [22]. Specimens were compared over the range 15.9–136 nmol/L. The average Error Index (Y-X)/TEa was −0.22 with a range of −1.02 to 0.86. The largest Error Index occurred at a concentration of 83.6 nmol/L. Deming regression analysis yielded a slope of 0.85 (95% CI, 0.79–0.91) and y-intercept of 3.11 nmol/L (95% CI, 0.07–6.16) (Fig. 1a).

We also analysed 69 specimens for 25OHD (old generation, 3L52) by Architect at Biomnis Ireland and by LC-MS/MS at St. James's Hospital, Dublin. The difference between the methods was within the allowable total error for 67 of 69 samples (97.1%). Specimens were compared over the range 27.8–170 nmol/L The average Error Index (Y-X)/TEa was 0.19 with a range of −0.52 to 1.63. The largest Error Index occurred at a concentration of 44.6 nmol/L. Deming regression analysis yielded a slope of 0.96 (95% CI, 0.88–1.04) and y-intercept of 6.73 nmol/L (95% CI, 1.11–12.35) (Fig. 1b).

**Fig. 2:**
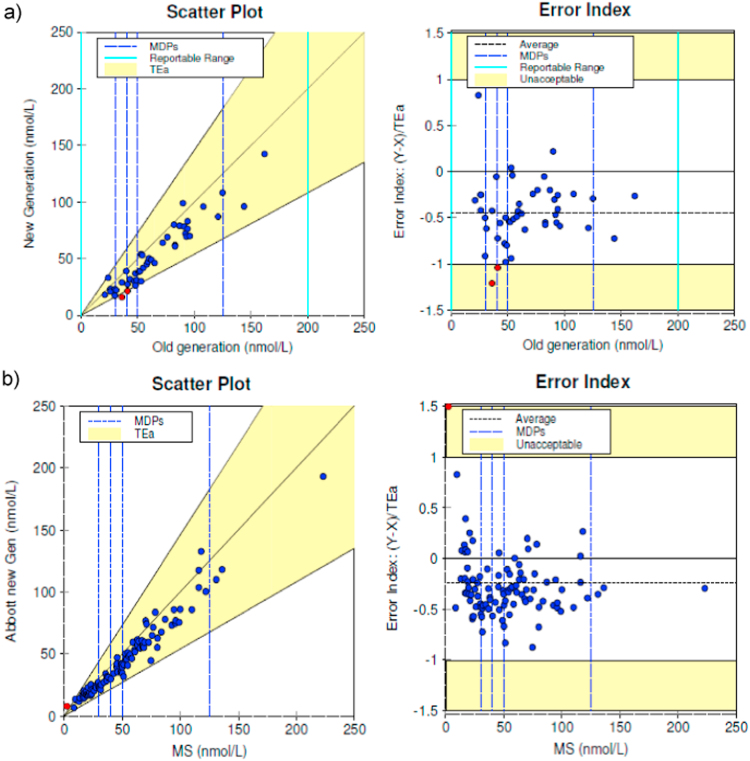
Two instrument comparisons between (a) the new (5P02) and old (3L52) generation of Architect 25OHD methods and (b) the new Architect 25OHD and LC-MS/MS(MS). The regression line for 47 samples (a), calculated according to Deming, is indicated (25OHD new generation nmol/L =0.87 × (25OHD old generation) nmol/L −4.9 nmol/L, r^2^=0.95). The regression line for 100 samples (b), calculated according to Deming, is indicated (25OHD new generation nmol/L =0.88 x (total 25OHD MS) nmol/L −1.2 nmol/L, r^2^=0.98). The difference between the two methods was within total allowable error (TEa) of 46%. The methods produced clinically equivalent results. Results covered the medical decision points (MDPs) of 30, 40, 50 and 125 nmol/L.

The new generation of 25OHD (5P02) was compared with the LC-MS/MS method using 100 patients’ samples. Only 6 of them had a significant 25(OH)D2 level. It should be noted that only total 25OHD levels were evaluated. Specimens were compared over the range 2.5–223 nmol/L. The difference between the two methods was within allowable error for 99 of 100 specimens (99%). The average Error Index (Y-X)/TEa was −0.24 with a range of −0.88 to 4.70. The largest Error Index occurred at a concentration of 2.5 nmol/L. Deming regression analysis yielded a slope of 0.88 (95% CI, 0.847–0.92) and y-intercept of −1.20 nmol/L (95% CI, −3.52−1.12). (Fig. 2b). We identified the range of data for which the difference was significant. Using Paired Sample Wilcoxon Signed Rank test we demonstrated that for a concentration less than 30 nmol/L the difference between the two methods was not significant (p=0.05). The difference between the two methods was significant at concentrations greater than 30 nmol/L.

The difference between the new and old generations of 25OHD Architect assays for 45 of 47 (96%) samples was within Allowable Total Error. Specimens were compared over the range of 21–162 nmol/L. The average Error Index (Y-X)/TEa was −0.45 with a range of−1.23 to 0.82. The largest Error Index occurred at a concentration of 55 nmol/L. Deming regression analysis yielded a slope of 0.87 (95% CI, 0.8−0.96) and y-intercept of −4.9 nmol/L (95% CI, −10.93−1.15) (Fig. 2a). Therefore, the old and new methods were shown not to be statistically equivalent (95% CI of slope does not include unity). However, all the instruments and methods produced clinically equivalent results. The results covered the medical decision points of 30, 40, 50 and 125 nmol/L. These decision points are based on the cutoffs for deficiency, inadequacy, adequacy and risk of excess [24], [25], [26] (Fig. 1, Fig. 2).

Both Abbott methods showed an excellent within-run imprecision, giving an uncertainty of measurement of 0.335 for the old generation and 0.266 for the new generation. The between-run uncertainties were 0.28 and 0.36, with expanded uncertainties 0.87 and 0.98 (k=2) for Old (3L52) and New (5P02) respectively. At a 25OHD concentration of 67 nmol/L the uncertainty of measurement was 0.87 nmol/L or 1.3% for the old generation. We can also say that we are 95% confident that the Vitamin D result is between 66 and 68 nmol/L for this value. For the new generation of Abbott reagents, at a 25OHD concentration of 57 nmol/L the uncertainty of measurement was 0.98 nmol/L or 1.72%, and we are 95% confident that the 25OHD result is between 55 and 59 nmol/L. (Table 1).

The new generation of the reagent shows an acceptable level of performance in RIQAS Immunoassay external quality assessment (Standard Deviation Index =−0.21(<2)) [27].

To establish our reference range, we analysed serum samples from 210 healthy adults (Irish Caucasians, age range: 29–65 years) for 25OHD on Abbott Architect. Participants currently using medications that modulate or influence vitamin D metabolism were excluded. Medical history, including consumption of alcohol and tobacco, was assessed by the nurse.

**Fig. 3:**
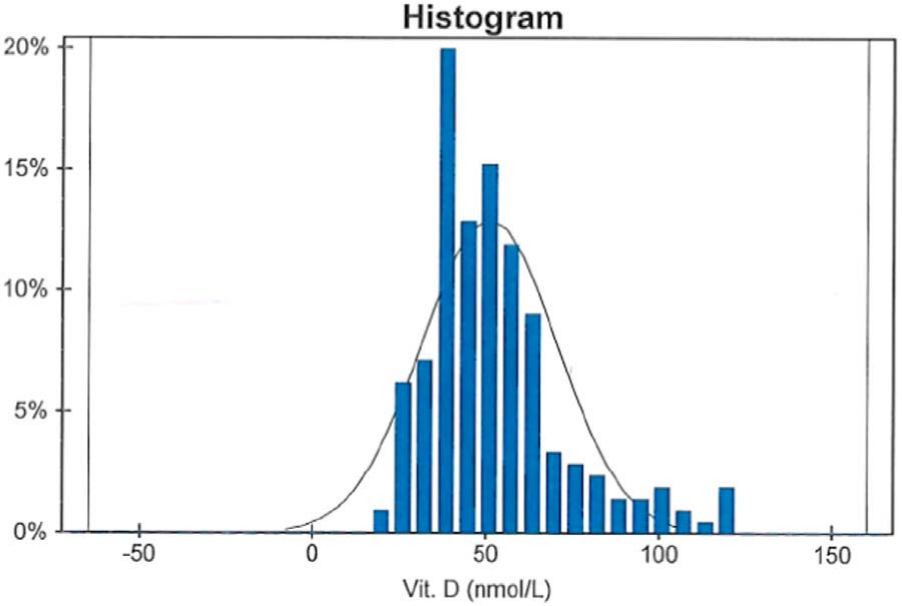
Reference interval data and distribution for 25OHD concentrations measured with the old generation of Abbott reagents (3L52). Results are from 210 healthy volunteers who provided serum samples for the study. No significant difference was observed in sex or age. All adults were Irish Caucasian. Distribution shows the non-Gaussian trend in the data, and median total 25OHD value for all subjects was 49 nmol/L.

**Fig. 4:**
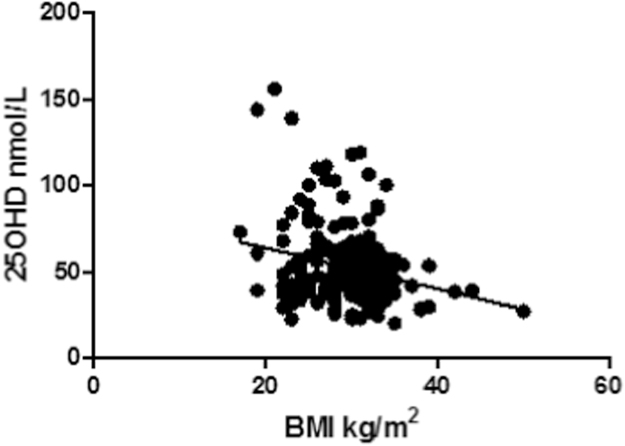
Correlation of patients’ body mass index (BMI) with 25OHD serum concentrations for 210 healthy adults (BMI mean =29.6 kg/m^2^±4.4; slope =−1.19; 95% confidence interval: −1.84 to −0.53; p=0.0005, r^2^=0.06).

The distribution of results for 25OHD was non-Gaussian. The calculated reference interval based on the central 95% of the data was 24 –111 nmol/L for total 25OHD. The median, 10th, and 90th percentiles of total 25OHD for all subjects were 49, 23, and 100 nmol/L, respectively. We observed no statistically significant differences as a result of sex or age (Fig. 3). But we found a significant negative correlation between BMI and 25OHD levels.

(r^2^ =0.06, p=0.0005) (Fig. 4). A high proportion of our patients were overweight (mean BMI =29.6 kg/m^2^).

The current decision intervals or cutoffs for 25OHD used at our institution based on dietary reference intakes [24], [25], [26] are as follows:

**Table:** 

Increased risk of deficiency:	< 30 nmol/L
Increased risk of inadequacy:	< 40 nmol/L
Adequacy:	> 50 nmol/L
Increased risk of excess:	> 125 nmol/L

Based on the newly proposed IOM definition of vitamin D sufficiency (>50 nmol/L), 51% of the healthy volunteers in our study had vitamin D deficiency.

## 4 Discussion

In this study, we verified the Abbott Architect total 25OHD vitamin D assays on the i2000SR platform. The old generation kit showed excellent CVs, and a comparison study with Diasorin Liaison and LC-MS/MS shows clinically equivalent results. Although the old generation Architect Vitamin D assay performed adequately there was potential to improve reagent and calibrator stability, improve alignment to LC-MS/MS (especially at high levels), and improve precision at low concentrations. The new generation of the Architect Vitamin D assay (5P02) shows many advantages over the old (3L52). These include: improved calibration stability from 7 to 30 days, one position used on reagent carousel vs two, 200 tests analysed per hour vs 100, increased reagent on board stability from 14 to 21 days, and improved imprecision – CV of 20% at approximately 6 nmol/L vs 20 nmol/L [28].

Although the Deming slope value obtained for the new generation assay indicates that the old assay aligns well with the LC-MS/MS method, it should be pointed out that the correlation coefficient for the new generation assay was higher (0.98). In addition, the correlation with the new assay also showed a lower intercept compared to the old assay and only one concentration value outside the TEa at a very low concentration of 2.5 nmol/L. This sample concentration falls below the Limit of Quantitation of the assay (6 nmol/L) [28]. Overall the correlation plot demonstrated a well-aligned distribution across and beyond the clinically relevant range.

The new assay is standardized to NIST SRM 2972 and VDSP certified from the Centres of Disease Control and Prevention. For our busy clinical laboratory the throughput of the new improved generation of 25OHD assays is a major advantage. We have significantly improved the turn-around-time for our patients for total 25OHD assays.

Our results are in line with a recent publication by Cavalier et al. [29] which provides a clinical and analytical evaluation of the same Abbott method. This study also shows a comparison of this method with a VDSP-traceable LC-MS/MS in six different populations and addressed cross-reactivity with vitamin D2.

One limitation of our investigations is that we did not perform cross-reactivity studies because only a small number (6%) of the samples had elevated 25(OH)D2 levels. Therefore we could not confirm Abbott cross-reactivity data. It is well recognised that mass spectrometry can resolve vitamin D2 completely from D3, allowing quantitation of the D2 form. However, immunoassays only partially detect vitamin D2, with varying degrees of cross-reactivity. This may not be important in Ireland, due to the limited food sources of vitamin D2 and the predominant use of vitamin D3 in supplementation.

Our Laboratory is accredited to ISO 15189, and ISO 15189, 5.6.2 requires that “The laboratory shall determine the uncertainty of results where relevant and possible”. Uncertainty of Measurement provides a quantitative estimate of the quality of a test result, and therefore is a core element of a quality system for clinical laboratories [30]. We have estimated the between-run uncertainty and expanded uncertainty for total 25OHD Architect methods [19]. Measurement uncertainty must be considered for an optimal interpretation of the measured values. This means that the “true” vitamin D concentration of a patient whose measured value is 50 nmol/L, for example, could be between 45 and 55 nmol/L, with a maximum analytical CV of 10%. We believe that measurement uncertainty must be taken into account at any cut-off level, and if we want to ensure that a measured 25OHD concentration is really >50 nmol/L, a value of at least 55–65 nmol/L should be targeted. We can say from our results that we have achieved an “ideal uncertainty” of measurement because our calculated UOM of 1.3% and 1.72% is <0.25 x CVw (2.5) [22].

Results from our reference interval study advocate the use of 25OHD guidelines based on disease states as opposed to reference intervals based on an apparently healthy population.

We believe that the strength of our study lies in the careful selection of participants without any chronic conditions who had normal results for calcium, phosphate, renal and liver function. A limitation is that, for financial reasons, we did not measure PTH levels. It is noteworthy that we found a negative correlation between 25OHD and BMI. We appreciate that obesity is a growing problem in Ireland and it is possible that it is one of the major factors in vitamin D deficiency.

There have been several hypotheses to explain the negative correlation between BMI and 25OHD levels. Some of them speculated that individuals with a higher BMI may engage in fewer outdoor activities due to their restricted mobility. They may also expose less skin to the sun, since they tend to cover themselves more than leaner individuals, thereby limiting endogenous production of 25OHD in the skin [31], [32]. It also may be explained by the storage of 25OHD in adipose tissue because of its high lipid solubility, leading to lower bioavailability in individuals with a higher BMI [33], [34].

It is also critical to note that these studies were conducted during the winter in Dublin, Ireland (latitude 53°N). A minimum energy of 20 kJ/cm^2^ is needed to produce cutaneous vitamin D3, and during the winter months in Ireland such an exposure level cannot be achieved, even during (rare) sunny conditions [35]. Thus our patients were at their lowest annual vitamin D status. Adequate levels >50 nmol/L were achieved only in 49% of healthy Irish volunteers. This percentage would be considerably smaller if the target range was raised to 75–100 nmol/L as suggested by some groups [36]. The calculated reference interval mean for total 25OHD for our subjects was only 49 nmol/L. Some clinicians may consider that vitamin D supplementation, without prior (expensive) determination of serum concentration, should be initiated in people at higher risk of deficiency, based on our findings. But we emphasise the importance of patients’ baseline vitamin D status. Patients with chronic illnesses may have different vitamin D metabolisms, and after supplementation it would be important to achieve sufficient levels of 25OHD to prescribe the correct dosage and achieve optimal health outcome.

Along with others [37], we believe that dominant 25OHD levels may be crucial, because they affect local tissue concentrations of the active vitamin D form [38]. Serum 25OHD levels up to 120 nmol/L may be necessary to achieve optimal immune function [39]. It has been claimed [39] that the anti-inflammatory benefit of vitamin D was seen only in patients whose 25OHD concentration reached >100 nmol/L. There were no beneficial effects when vitamin D status fell to below 100 nmol/L.

From our findings we conclude that, in Ireland, and especially during the winter months, vitamin D testing could be important for general health screening. More study is warranted to establish reference values that truly represent the healthy Irish population, including seasonal variations.

## Conflicts of interest

None declared.

## Funding

This paper was supported by Research Grant from the Irish Research Council. Ethical approval: study samples were used from the trial registered at ClinicalTrials.gov (Identifier: NCT02428322).

## Guarantor

KH.

## Contributorship

All authors were involved in the design of the research. KH and MH performed laboratory analysis and collated data. KH wrote the first draft of the manuscript.

All authors reviewed and edited the manuscript, analysed the data and approved the final version of the manuscript.

## References

[1] Holick M.F. (2007). Vitamin D deficiency. N. Engl. J. Med..

[2] Kerley C.P., Elnazir B., Faul J., Cormican L. (2015). Vitamin D as an adjunctive therapy in asthma. Part 2: a review of human studies. Pulm. Pharm. Ther..

[3] Pilz S. (2009). Vitamin D status and arterial hypertension: a systematic review. Nat. Rev. Cardiol..

[4] Holick M.F. (2009). Vitamin D status: Measurement, interpretation, and clinical application. Ann. Epidemiol..

[5] Heaney R.P. (2011). Serum 25-hydroxyvitamin D is a reliable indicator of vitamin D status. Am. J. Clin. Nutr..

[6] Hollis B.W. (2010). Assessment and interpretation of circulating 25-hydroxyvitamin D and 1,25-dihydroxyvitamin D in the clinical environment. Endocrinol. Metab. Clin. North Am..

[7] Ross A.C., Manson J.E., Abrams S.A., Aloia J.F., Brannon P.M., Clinton S.K. (2011). The 2011 report on dietary reference intakes for calcium and vitamin D from the Institute of medicine: what clinicians need to know. J. Clin. Endocrinol. Metab..

[8] Heaney R.P., Holick M.F. (2011). Why the IOM recommendations for vitamin D are deficient. J. Bone Min. Res..

[9] Holick M.F. (2011). The IOM D-lemma. Public Health Nutr..

[10] Wallace A.M., Gibson S., de la Hunty A., Lamberg-Allardt C., Ashwell M. (2010). Measurement of 25-hydroxyvitamin D in the clinical laboratory: current procedures, performance characteristics and limitations. Steroids.

[11] Bailey D., Veljkovic K., Yazdanpanah M., Adeli K. (2013). Analytical measurement and clinical relevance of vitamin D(3) C3-epimer. Clin. Biochem..

[12] Keevil B. (2012). Does the presence of 3-epi-25OHD 3 affect the routine measurement of vitamin D using liquid chromatography tandem mass spectrometry?. Clin. Chem. Lab Med..

[13] Enko D., Fridrich L., Rezanka E., Stolba R., Ernst J., Wendler I. (2014). 25-hydroxy-Vitamin D status: limitations in comparison and clinical interpretation of serum-levels across different assay methods. Clin. Lab.

[14] Fraser W.D., Milan A.M. (2013). Vitamin D assays: past and present debates, difficulties, and developments. Calcif. Tissue Int..

[15] Farrell C.J.L., Martin S., McWhinney B., Straub I., Williams P., Herrmann M. (2012). State-of-the-Art Vitamin D Assays: a comparison of automated immunoassays with liquid chromatography–tandem mass spectrometry methods. Clin. Chem..

[16] Holmes E.W., Carbincius J., McKenna K.M. (2013). Analytical variability among methods for the measurement of 25-hydorxyvitamin D. Am. J. Clin. Pathol..

[17] Wielders J.P., Wijnberg F.A. (2009). Preanalytical stability of 25(OH)-vitamin D3 in human blood or serum at room temperature: solid as a rock. Clin. Chem..

[18] NCCLS EP5-A2 (2004). Evaluation of Precision Performance of Quantitative Measurement Methods.

[19] Bell S. (1999). Measurement Good Practice Guide No.11. A Beginner's Guide to Uncertainty of Measurement.

[20] Thienpont L.M. (2008). Calculation of measurement uncertainty—Why bias should be treated separately. Clin. Chem..

[21] NCCLS. How to define and determine reference intervals in the clinical laboratory, Approved Guideline-second edition. Wayne, PA, NCCLS document C28-A2, 2000.

[22] Stockl D., Sluss P.M., Thienpont L.M. (2009). Specifications for trueness and precision of a reference measurement system for serum/plasma 25-hyrodroxyvitamin D analysis. Clin. Chim. Acta.

[23] DEQAS: Vitamin D External Quality Assessment Scheme. 〈http://www.deqas.org/〉.

[24] Holick M.F., Binkley N.C., Bischoff-Ferrari H.A., Gordon C.M., Hanley D.A., Heaney R.P. (2011). Evaluation, treatment, and prevention of vitamin D deficiency: an Endocrine Society Clinical Practice Guideline. J. Clin. Endocrinol. Metab..

[25] Aloia J.F. (2011). The 2011 report on dietary reference intake for vitamin D: where do we go from here?. J. Clin. Endocrinol. Metab..

[26] (2011). Committee to Review Dietary Reference Intakes for Vitamin D and Calcium & Institute of Medicine. Dietary Reference Intakes for Calcium and Vitamin D.

[27] RIQAS Evaluation of Performance. 〈http://www.riqasconnect.randox.com/〉.

[28] (2015). Abbott Diagnostics. Architect 25-OH Vitamin D Instructions for Use.

[29] Cavalier E., Lukas P., Bekaert A.C., Carlisi A., Le Goff C., Delanaye P., Souberbielle J.C. (2016). Analytical and clinical validation of the new Abbot Architect 25(OH)D assay: fit for purpose?. Clin. Chem. Lab Med..

[30] White G.H., Farrance I. (2004). Uncertainty of measurement in quantitative medical testing – a laboratory implementation guide. Clin. Biochem. Rev..

[31] Compston J.E., Vedi S., Ledger J.E., Webb A., Gazet J.C., Pilkington T.R. (1981). Vitamin D status and bone histomorphometry in gross obesity. Am. J. Clin. Nutr..

[32] Pourshahidi L.K. (2015). Vitamin D and obesity: current perspectives and future directions. Proc. Nutr. Soc..

[33] Need A.G., Morris H.A., Horowitz M., Nordin C. (1993). Effects of skin thickness, age, body fat, and sunlight on serum 25-hydroxyvitamin D. Am. J. Clin. Nutr..

[34] Wortsman J., Matsuoka L.Y., Chen T.C., Lu Z., Holick M.F. (2000). Decreased bioavailability of vitamin D in obesity. Am. J. Clin. Nutr..

[35] Webb A.R., Kline L., Holick M.F. (1988). Influence of season and latitude on the cutaneous synthesis of vitamin D3 synthesis in human skin. Arch. Dermatol..

[36] Souberbielle J.C., Body J.J., Lappe J.M., Plebani M., Shoenfeld Y., Wang T.J. (2010). Vitamin D and musculoskeletal health, cardiovascular disease, autoimmunity and cancer: recommendations for clinical practice. Autoimmune Rev..

[37] Zhang Y., Leung D.Y., Richers B.N., Liu Y., Remigio L.K., Riches D.W. (2012). Vitamin D inhibits monocyte/macrophage proinflammatory cytokine production by targeting MAPK phosphatase-1. *J*. Immunol..

[38] Hewison M. (2011). Antibacterial effects of vitamin D. Nat. Rev. Endocrinol..

[39] Ojaimi S., Skinner N.A., Strauss B.J., Sundararajan V., Woolley I., Visvanathan K. (2013). Vitamin D deficiency impacts on expression of toll-like receptor-2 and cytokine profile: a pilot study. J. Transl. Med..

